# Extracellular Matrix Optimization for Enhanced Physiological Relevance in Hepatic Tissue-Chips

**DOI:** 10.3390/polym13173016

**Published:** 2021-09-06

**Authors:** Abdul Rahim Chethikkattuveli Salih, Kinam Hyun, Arun Asif, Afaque Manzoor Soomro, Hafiz Muhammad Umer Farooqi, Young Su Kim, Kyung Hwan Kim, Jae Wook Lee, Dongeun Huh, Kyung Hyun Choi

**Affiliations:** 1 Department of Mechatronics Engineering, Jeju National University, Jeju-si 63243, Korea; abdulrahim@jejunu.ac.kr (A.R.C.S.); gusrlska04@gmail.com (K.H.); arunasif@hotmail.com (A.A.); umerfarooqi@jejunu.ac.kr (H.M.U.F.); rudghks624@gmail.com (K.H.K.); jaewook482@gmail.com (J.W.L.); 2 Department of Electrical Engineering, Sukkur IBA University, Airport Road, Sukkur 65200, Pakistan; afaquemanzoor@gmail.com; 3 BioSpero, Inc., Jeju-si 63243, Korea; youngsu1742@gmail.com; 4 Department of Bioengineering, University of Pennsylvania, Philadelphia, PA 19104, USA; huhd@seas.upenn.edu

**Keywords:** extracellular matrix, microphysiological system, TEER, tight junction proteins, image analysis, collagen

## Abstract

The cellular microenvironment is influenced explicitly by the extracellular matrix (ECM), the main tissue support biomaterial, as a decisive factor for tissue growth patterns. The recent emergence of hepatic microphysiological systems (MPS) provide the basic physiological emulation of the human liver for drug screening. However, engineering microfluidic devices with standardized surface coatings of ECM may improve MPS-based organ-specific emulation for improved drug screening. The influence of surface coatings of different ECM types on tissue development needs to be optimized. Additionally, an intensity-based image processing tool and transepithelial electrical resistance (TEER) sensor may assist in the analysis of tissue formation capacity under the influence of different ECM types. The current study highlights the role of ECM coatings for improved tissue formation, implying the additional role of image processing and TEER sensors. We studied hepatic tissue formation under the influence of multiple concentrations of Matrigel, collagen, fibronectin, and poly-L-lysine. Based on experimental data, a mathematical model was developed, and ECM concentrations were validated for better tissue development. TEER sensor and image processing data were used to evaluate the development of a hepatic MPS for human liver physiology modeling. Image analysis data for tissue formation was further strengthened by metabolic quantification of albumin, urea, and cytochrome P450. Standardized ECM type for MPS may improve clinical relevance for modeling hepatic tissue microenvironment, and image processing possibly enhance the tissue analysis of the MPS.

## 1. Introduction

Organs on a chip (OoC) technology is based on providing a dynamic microenvironment with physiological shear stress for optimum growth and differentiation of tissues emulating human physiology [1]. Microphysiological systems (MPS) are used for studying drug toxicities, disease modeling, and reverse engineering of human organs. Biocompatible materials and porous membranes support the cellular scaffolds in MPS [2]. Cells are suspended in hydrogels or attached to a surface with supporting extracellular matrix (ECM). ECM interactions influence cell adhesion, cell differentiation, cell–cell communication, tissue repair, tissue regeneration, and tumor growth processes [3]. Cell isolation from native ECM causes loss of cellular polarity and important molecular characteristics [4]. Commercially available ECM components are typically employed as cell culture substrata. Exclusive ECM components, such as fibronectins, collagens, and laminins have been utilized in cell culture for years and have been proved to profoundly impact the survival and attachment of cells cultured in vitro, and homeostasis of various cellular functions [5].

Standardization of the MPS for obtaining approval from regulatory bodies has become crucial [6]. Issues concerning cell culture in MPSs, such as cell number, cell type, tissue-specific ECM, and standard biomarker testing methods, need to be standardized for emulating human physiology [7]. Salih et al. studied the effect of serum concentration on tight junction protein within MPSs by using a TEER sensor, which highlighted the direct influence on tight junction proteins (TJPs) required for attachment and biomarker production [8]. Accumulating evidence has indicated the positive impact of MPS surfacing modification by ECM relevant for a specific tissue type [9,10,11]. In addition, ECM influences the maintenance of pluripotent stem cells (PSCs) and plays an important role in PSC differentiation [12]. In particular, the attachment proteins required for adherence of a tissue to a specific ECM must be defined with respect to each organ [13,14].

Fiji, an image processing package based on ImageJ, is used to perform image thresholding to evaluate multiple features of cell culture, mainly cell confluency. The TEER sensor has indicated a potential to measure tight junction formation and deformation. Additionally, LabVIEW with IMAQ Vision tools has the potential for image processing data. Previously, we highlighted the use of LabVIEW-based assessment of ROS production in an MPS with an integrated microscope [15,16,17,18]. Color intensity-based processing of 2D and 3D images generated from histograms generated through IMAQ assists in image data analysis. Histogram-based peaks of pixel intensity present a reliable assessment of tissue formation when utilized for cell culture staining images. Previously, constitutive equations have been utilized for predicting the material behavior conditions. In addition, constitutive equations have the potential to assist in indicating outcomes based on a polynomial regression model for ECM. There is a lack of consensus for the selection of ECM to perform in vitro cell culture assays on MPS platforms.

**Figure 1:**
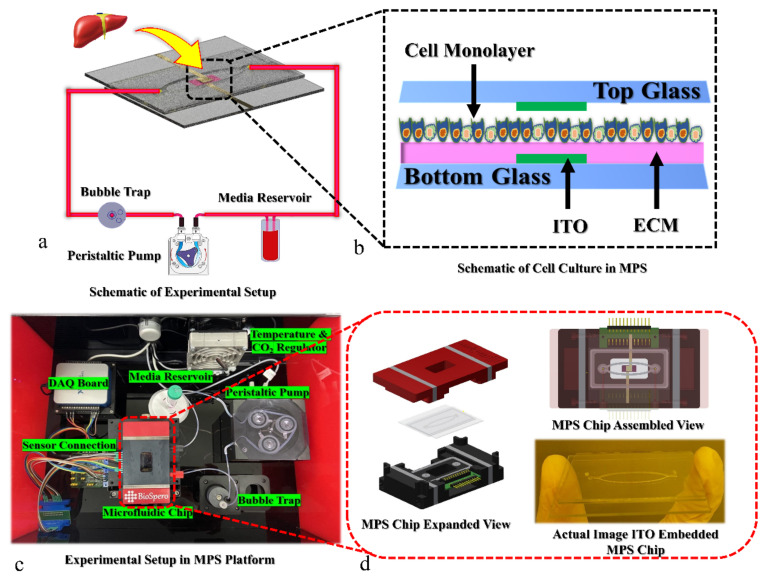
Schematic representation of hepatic MPS platform setup. (**a**) Schematic representation of experimental setup, hepatic tissue culture in dynamic environment with TEER sensor representation. (**b**) Magnified view of hepatocyte culture on ECM-coated glass chip with ITO TEER sensor. (**c**) Real image of experimental setup, top view of engineered MPS environment, a microfluidic chip with embedded TEER sensor for tight junction evaluation is connected to the media reservoir with tubing in which medium is circulated using peristaltic pump and a bubble trap is attached to the system for bubble removal. Sensor control unit, temperature controller, and CO_2_ regulator maintain cell culture incubation conditions. (**d**) MPS chip exploded view, MPS chip assembled view with connected transducers and the actual image of ITO embedded MPS chip.

In the present study, we evaluated the impact of different singular types of commercially available ECM on a liver MPS in comparison with Matrigel^TM^—a mixture of ECM components, with Figure 1 representing a schematic. Several concentrations of different ECM types (collagen, fibronectin, and poly-L-lysine) were tested for cell attachment and tissue growth in comparison with different concentrations of Matrigel. We utilized an image analysis technique based on image thresholding and implemented a statistical model to analyze cell attachment and confluency development. The current application of mathematical models has the potential to predict cell attachment with respect to ECM concentration. Furthermore, rigorous image analysis approaches were utilized to identify the optimum ECM type and concentration. These ECM concentrations were then used in a dynamic cell culture environment with a TEER sensor, and several biological parameters were studied with respect to the liver MPS. The metabolic profiles of molecular biomarkers presented a vague assessment of tissue formation compared to that of image processing. We utilized the LabVIEW tool for investigating the total green intensity graph of the image, green intensity of tight junction protein, albumin staining, and live/dead assay. This may infer that specific ECM modulations improve tissue quality and image analysis tools may support the conventional assays’ results. Additionally, the image analysis tool can be used for biomarker analysis through fluorescence staining images.

## 2. Materials and Methods

### 2.1. Microfluidic Chip Fabrication

To establish a microfluidic chip, two soda lime glass chips were utilized by stacking them. Glass chips with a thickness of 1.1 mm, width of 41 mm, and length 56 mm were taken and polydimethylsiloxane (Sylgard 184, Dow corning, Bristol, PA, USA) was fixed using plasma treatment to form the microfluidic channels. The height of the microfluidic channels was 300 µm and the width of the channel was 800 µm. A magnetic chip holder was designed to assemble the glasses in the form of a microfluidic chip.

### 2.2. Analysis of the Effect of ECM on Liver Cells

Four different ECM types were used to study the impact of ECM on adherence of cells to the surface of the glass chip. Collagen type I (rat tail) (Sigma-Aldrich, Burlington, MA, USA) was used in different concentrations of 100, 125, 150, 175, and 200 µg/mL in PBS (Thermo Fisher Scientific, Waltham, MA, USA). Poly-L-lysine (1 mg/mL) (ScienCell, Carlsbad, CA, USA) was diluted in sterile double distilled water to obtain concentrations of 2, 3, 5, 6, and 7 µg/mL. Fibronectin (Thermo Fisher, Waltham, MA, USA) solution with distilled H_2_O was prepared at 1 mg/mL concentration which was serially diluted in PBS to obtain concentrations of 10, 13, 15, 20, and 25 µg/mL. Matrigel (Corning Inc., Corning, NY, USA) was thawed overnight in ice diluted with cold Dulbecco’s modified Eagle medium (DMEM) to desired concentrations of 100 µg/mL, 125 µg/mL, 150 µg/mL, 175 µg/mL, and 200 µg/mL. For all ECM types, the volume for coating was fixed at 400 µL before cell seeding in the microfluidic chips followed by an overnight incubation in a cell culture incubator.

### 2.3. Cell Seeding and Development of Liver MPS

The liver epithelial cell line HepG2 (KCLB No. 88065) derived from human hepatoblastoma was purchased from Korean Cell Line Bank, Korean Cell Line Research Foundation, Seoul, South Korea. The immortal HepG2 cells were cultured in 10% fetal bovine serum containing DMEM cell culture media supplemented with 1% *v*/*v* penicillin and streptomycin (P/S) antibiotic solution and incubated at 37 °C with 5% CO_2_ [19]. Before starting the experiment, glass chips were sterilized using 70% ethanol and a subsequent ultraviolet exposure for 1 h.

Firstly, the glass chip was fixed in a magnetic seeding kit and ECM was coated to provide an improved adherence surface for cell culture. ECM coating was performed using 4 types of material, i.e., solutions of Matrigel, fibronectin, collagen, and poly-L-lysine. After ECM coating, chips were incubated for 4 h at 37 °C to create an ECM bed for cell culture in the MPS before seeding the cells. Before seeding on the chip, hepatocytes were cultured for 2 doublings. HepG2 cells were seeded at the density of 2 × 10^5^ cells/500 µL. Again, overnight incubation in a cell culture incubator was performed after seeding the cells on glass chips for adequate attachment on the surface. The seeding kit after cell seeding was covered with sealing membrane (Breathe-Easy^®^, Sigma-Aldrich, Burlington, MA, USA) (Figure S1).

Once the cells had been attached on the glass surface, the culture medium was removed, and the glass chip was placed in a magnetic chip holder to form a hepatic MPS (M-Physio™ Platform, Biospero, Inc, Korea). This MPS functioned by attaching a micropump with tubing and conditions were kept at 37 °C and 5% CO_2_ to facilitate the cell culture conditions. An amber colored 15 mL Falcon tube was attached to the MPS using tubing connection with a peristaltic pump. The volume of cell culture media was kept at 7 mL and changed every 2 days. The MPS shear stress was calculated to be 0.5 dyn/cm^2^ and, based on this requirement, medium flow rate was set at 60 μL/min. Calculation of shear stress is shown in Equation (1):

(1) $$\tau =\frac{6µQ}{wh^{2}}$$

where “*h*” indicates the height of the channel, “*w*” represents the width, “*Q*” represents the flow rate of the media, and “μ” represents viscosity of the cell culture medium. The cell chamber was 9 mm in width, 200 µm in height, 60 μL/min with viscosity of 0.81 mPas.

### 2.4. Measurement of CYP450, Urea, and Albumin Enzyme Levels

The levels of functional biomarkers of hepatocytes, including urea, albumin, and cytochrome P450 enzyme activity, were measured using a Human Albumin ELISA Kit (Abcam, Cambridge, UK), Urea Kit (Abnova, Taipei City, Taiwan), and P450-Glo CYP3A4 Assay Kit (ca# V9001, Promega, Madison, WI, USA). Medium samples collected at consecutive intervals were stored at −80 °C after centrifugation. Similarly, a CYP34A quantification assay was performed with the stored cell culture medium samples. Absorbance was measured by a multipurpose microplate reader (SpectraMax i3 Multimode Microplate Reader, Molecular Devices, San Jose, CA, USA).

### 2.5. Live/Dead Assay

Dulbecco’s PBS (DPBS) was used to wash the cell culture area of the microfluidic chips three times and a LIVE/DEAD Cell Viability Assay Kit (Thermo Fisher, Waltham, MA, USA) was used according to the manual. The microfluidic chips were then incubated in a humidified cell culture incubator at 5% CO_2_ at 37 °C for 30 min. The cell surface was rinsed with DPBS and mounted with Fluoromount Aqueous Mounting Medium (Sigma-Aldrich, Burlington, MA, USA), subsequently a coverslip was placed on stained tissue. A confocal laser scanning microscope (Olympus FV122, Olympus, Tokyo, Japan) was utilized at excitation wavelength of 530–560 nm and emission wavelength of 530–645 nm for obtaining fluorescent images. The confocal micrographs were processed for live and dead cell counting using ImageJ software (Version 1.52 p, National Institute of Health, USA).

### 2.6. ZO-1, E-cadherin, and Albumin Immunofluorescence Microscopy

ZO-1 immunofluorescence staining was carried out following the given instructions (primary ZO-1 antibodies, cat# 33–9100, Thermo Fisher, Waltham, MA, USA). The cell culture area of the microfluidic glass chips was washed three times using a pre-warmed 1 × DPBS solution. The microfluidic chips were incubated with chilled 70% ethanol for 5 min at room temperature and then with 4% paraformaldehyde (cat# AAJ19943K2, Thermo Fisher, Waltham, MA, USA) in DPBS for 10 min at 23 °C. The microfluidic chips were again incubated with 1% bovine serum albumin (BSA; cat# 15561020, Thermo Fisher, Waltham, MA, USA) for 60 min to block non-specific antibody binding. Later, the chips were rinsed twice with DPBS. The chips were then incubated overnight with secondary antibodies (goat anti-mouse IgG, cat# ab205719, Abcam, USA) diluted 1:50 in BSA at 4 °C. Subsequently, the microfluidic chips were rinsed three times with DPBS for 5 min in the dark. The cells were dyed for 3 min with 300 nM DAPI solution (4′, 6-Diamidino-2-Phenylindole, Dihydrochloride, cat# D1306, Thermo Fisher, Waltham, MA, USA) prepared in DPBS. For E-cadherin immunofluorescence staining, the manufacturer’s instructions were followed with slight modifications, and microfluidic chips were incubated with anti-E-cadherin antibodies (cat# M168-C-terminal ab76055, Abcam, USA) and a secondary antibody (goat anti-mouse IgG, cat# ab205719, Abcam, USA) in 1% BSA. After that, the cells were stained for 3 min with 300 nM DAPI solution.

### 2.7. Statistical Analysis

To validate the results, an image-based viability study was performed from different positions of the chip and the relative light unit (RLU) was calculated multiple times. To verify the statistical significance of the data, one-way analysis of variance (ANOVA) was performed using Tukey’s honestly significant difference (HSD) procedure which facilitates pairwise comparisons within the acquired data. For statistical comparisons, a *p* value ≤ 0.05 was considered significant and is denoted by “*”.

## 3. Results and Discussion

### 3.1. Cell Attachment and Image Analysis

Matrigel, fibronectin, collagen, and poly-L-lysine were applied at multiple concentrations for cell attachment and images were collected following incubation for 24 h, as shown in Figure S2. ECM components such as collagen and fibronectin have been previously used for the attachment of hepatocytes to a biocompatible surface or membranes [20,21,22]. While no standardized strategy has been formulated to use a specific ECM for liver MPS development, we systematically selected five common concentration ranges of ECM for attachment of hepatocytes, such as 100–200 µg/mL for collagen and Matrigel, 10–25 µg/mL for fibronectin and 2–7 µg/mL for poly-L-lysine. The cell attachment increased significantly on all four different types of ECM coatings with an increase in the concentration of each ECM within the range given above (100–200 µg/mL, 10–25 µg/mL, 2–7 µg/mL) It was found that the ECM concentration is directly proportional to the cell attachment (Table 1). These data showed that all ECMs increased hepatocyte attachment; however, there was no apparent tissue specificity observed at this stage.

The cell attachment ratios and cell confluency percentages were calculated using the image thresholding technique with the image analysis software Fiji. Matrigel was found to be the most effective ECM in preserving cell morphology and attachment to the glass surface, followed by fibronectin. However, cell attachment was lower with collagen and poly-L-lysine than that with Matrigel and fibronectin. As Matrigel is a commercially available ECM and constitutes multiple structural components of native ECM, it showed better tissue growth support compared to the other studied ECM types.

### 3.2. Mathematical Modeling and Confirmation of the Prediction Model

Based on the image analysis, a mathematical model was generated using a polynomial equation. Here, we used a regression model between the ECM concentration as output response ($P\left(x_{i}\right)$) and cell attachment as input variables ($x_{i}$).

(2) $$P\left(x_{i}\right)={\;p}_{0}+{\;p}_{1}x_{i}+{\;p}_{2}x_{i}{}^{2}+\cdots +{\;p}_{n}x_{i}{}^{n}+{\;f}_{i}$$

where ${\left\{p_{i}\right\}}_{i=0}^{n}$ are the coefficients of the regression model. Alternatively, Equation (4) can be rewritten in the matrix form as [23,24]

(3) $$\left(\begin{array}{c} P_{1}\\ P_{2}\\ \begin{array}{c} \vdots \\ P_{n} \end{array} \end{array}\right)=\left(\begin{array}{ccccc} 1 & x_{1} & x_{1}^{2} & \cdots & x_{1}^{n}\\ 1 & x_{2} & x_{2}^{2} & \cdots & x_{2}^{n}\\ \vdots & \vdots & \vdots & \ddots & \vdots \\ 1 & x_{n} & x_{n}^{2} & \ldots & x_{n}^{n} \end{array}\right)\left(\begin{array}{c} p_{0}\\ p_{1}\\ \begin{array}{c} \vdots \\ p_{n} \end{array} \end{array}\right)+\left(\begin{array}{c} f_{1}\\ f_{2}\\ \begin{array}{c} \vdots \\ f_{n} \end{array} \end{array}\right)$$

Equation (5) can be simplified into Equation (4) as:

(4) P=Xp+f

Here, P, f, p, and X represent measurement observations, measurement noise, regression coefficients, and input cell attachment, respectively, in matrix and vector forms. For estimating the regression coefficients of the polynomial in Equation (5), the least square method was used by performing error minimization between the original input and estimated points. The estimated coefficients following the least square method are:

(5) $$\hat{p}={\left(X^{T}X\right)}^{-1}X^{T}P$$

Incorporating estimated regression coefficients $\left(\hat{p}\right),$ the output ECM concentrations $\left(\hat{P}\right)$ for the unknown points can be obtained as:

(6) $$\hat{P}=X\hat{p}$$

A pattern of cell attachment percentage with respect to unknown concentrations of the relevant ECM was developed using the polynomial equation. A unique mathematical model was employed to determine the most reasonable values or concentrations of the ECM based on the available experimental data. Various metrics are available for the evaluation of the surrogate model accuracy. However, they require verification of the fitted surrogates. Hence, we examined the model adequacies by employing the coefficient of determination R^2^, root square error, and adjusted-R^2^. Here, R^2^ measured the variability in an observed response accounted for by the fitted surrogate model, ranging from 0 to 1. Ideally, a workable surrogate model should have a large R^2^ (in the range 0.95–1.00) (Equation (1)). Adjusted-R^2^ is the modified form of R^2^ adjusted for the number of input or control variables in the model. It is essential to evaluate the adjusted-R^2^, as it compensates the statistic based on the number of independent variables in the model (Equation (2)). The root mean square error (RMSE) quantifies the differences between the observed data and the data predicted by the surrogate. A closer fit concerning the observation presents a smaller value of Equation (3).

(7) $$R^{2}=\;1-\;\frac{\sum_{i}{\left(y_{i}-\hat{y}\right)}^{2}}{\sum_{i}{\left(y_{i}-\bar{y}\right)}^{2}}$$

(8) $$\mathrm{RMSE}=\sqrt{\frac{\sum_{i=1}^{n}{\left({\hat{y}}_{i}-y_{i}\right)}^{2}}{n}}$$

**Figure 2:**
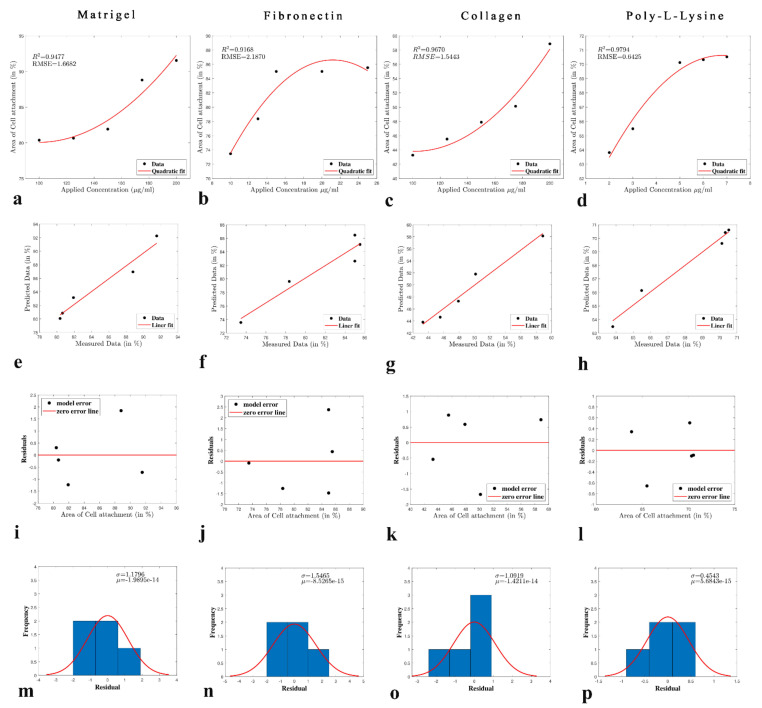
Mathematical model output obtained using MATLAB polynomial regression. (**a**–**d**) Correlation plot between cell attachment and ECM concentration for Matrigel, fibronectin, collagen, and poly-L-lysine, respectively (model fit). (**e**–**h**) R Square linear fit result of cell attachment and ECM concentration of Matrigel, fibronectin, collagen, and poly-L-lysine, respectively. (**i**–**l**) Residual plot result between cell attachment and ECM concentration for Matrigel, fibronectin, collagen, and poly-L-lysine, respectively. (**m**–**p**) Histogram plot for Matrigel, fibronectin, collagen, poly-L-lysine, respectively.

As shown in Figure 2, the prediction models were verified via re-experimentation with the selected ECM concentrations, as mentioned in Table 2 (Supplementary Materials, Figure S10 represents the code execution in MATLAB). The concentrations chosen were as follows: Matrigel, 120 µg/mL; fibronectin, 11 µg/mL; collagen, 130 µg/mL; and poly-L-lysine, 2.5 µg/mL. The difference between the re-experimentation results and the prediction models for each ECM was calculated using the polynomial Equation (4).

The coefficients (with 95% confidence bounds) are listed in Table 2. The predicted values were 1.662 for Matrigel, 3.183 for fibronectin, 2.383 for collagen, and 4.976 for poly-L-lysine. The implemented code for driving the abovementioned application of the polynomial regression model is given in Figure S10.

### 3.3. Microphysiological System Development

**Figure 3:**
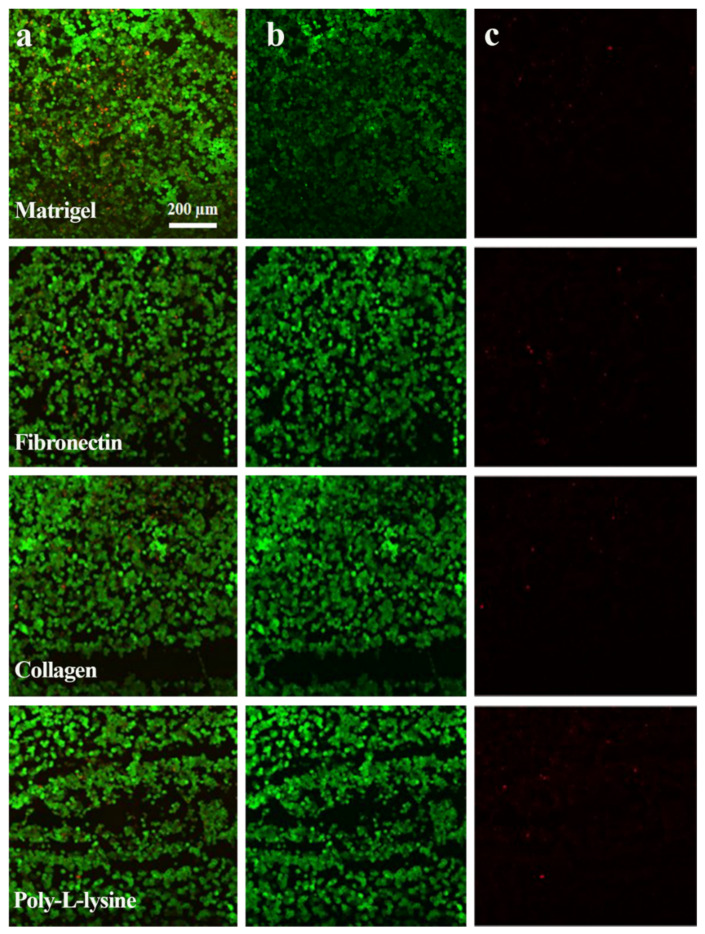
Live/Dead assay confocal images. Cell viability (live/dead assay) of HepG2 cell line microfluidic culture in different ECM substrata i.e., Matrigel, Fibronectin, Collagen, and Poly-L-Lysine. (**a**) Merge result of ethidium and Calcein-AM (**b**) live cell confocal images represented in green color (Calcein-AM) (**c**) The red color (ethidium) representing dead cells. Scale bar: 200 μm.

The statistical model developed by implementing the polynomial equation predicted the attachment percentage in a range of ECM concentrations. To verify the prediction method, random concentrations of ECM were selected to analyze the predictability of the mathematical model. We randomly selected one ECM concentration for each of the ECMs studied. Matrigel, collagen I, fibronectin, and poly-L-lysine were coated in the cell culture chamber at concentrations of 120 µg/mL, 130 µg/mL, 11 µg/mL, and 2.5 µg/mL, respectively. Table 2 gives an overview of the comparison attained from the randomly selected ECM values for their predicted and actual attachment capacities. It was found that the selected concentrations of the ECMs gave similar attachment results as predicted without significant differences. However, significant cell detachment from the microfluidic glass chip surface was observed using collagen and poly-L-lysine, as shown in Figure 3. Simultaneously, Matrigel and fibronectin were found to be more favorable for cell attachment.

### 3.4. TEER Assessment

**Figure 4:**
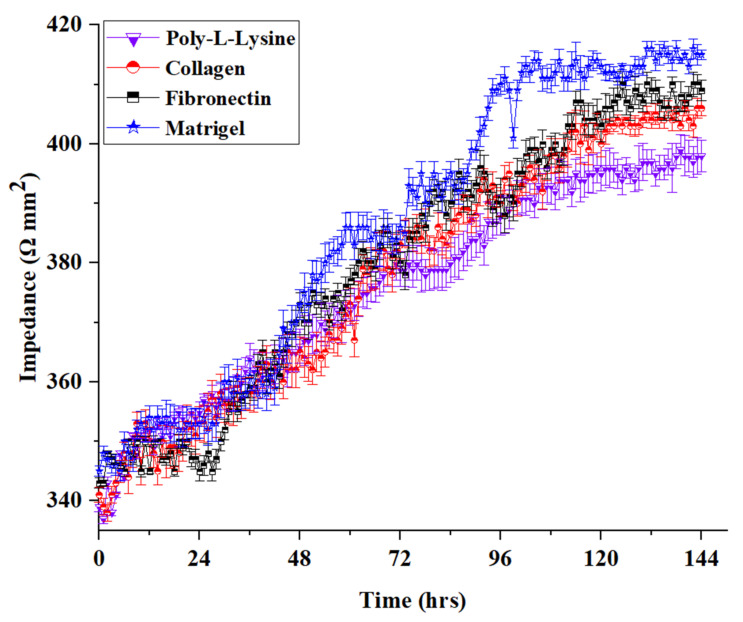
Real-time TEER data graph presenting the comparative impedance to different ECM time graphs in the liver MPS (data presented as mean ± SD). In supplementary data, each plot is shown separately (SF.2).

A previously validated TEER sensor was used as an additional parameter for assessing cell layer confluency in a chip platform [8,10,25,26]. The TEER sensor was used to assess the real-time effect of different ECMs on the barrier integrity of HepG2 cell monolayer formed in the MPS based on impedance monitoring for 144 h (Figure 4 and Figure S9). It was observed that ECM has a significant impact on the formation of tight junctions. TEER values among different ECMs have a substantial effect on the MPS-based real-time biological assays, as several researchers have employed TEER for estimating cell viability, fibrosis development, and FBS standardization [8,27,28,29]. It can be concluded that the choice of ECM is vital for developing the most physiologically relevant MPSs. The Matrigel-based liver MPS showed the highest TEER values compared to the remaining ECMs. This can be attributed to the higher molecular weight and better cell attachment of the liver cells on Matrigel than on other ECMs. The lowest TEER values were observed with poly-L-lysine.

### 3.5. Expression of Tight Junction Protein in MPS

**Figure 5:**
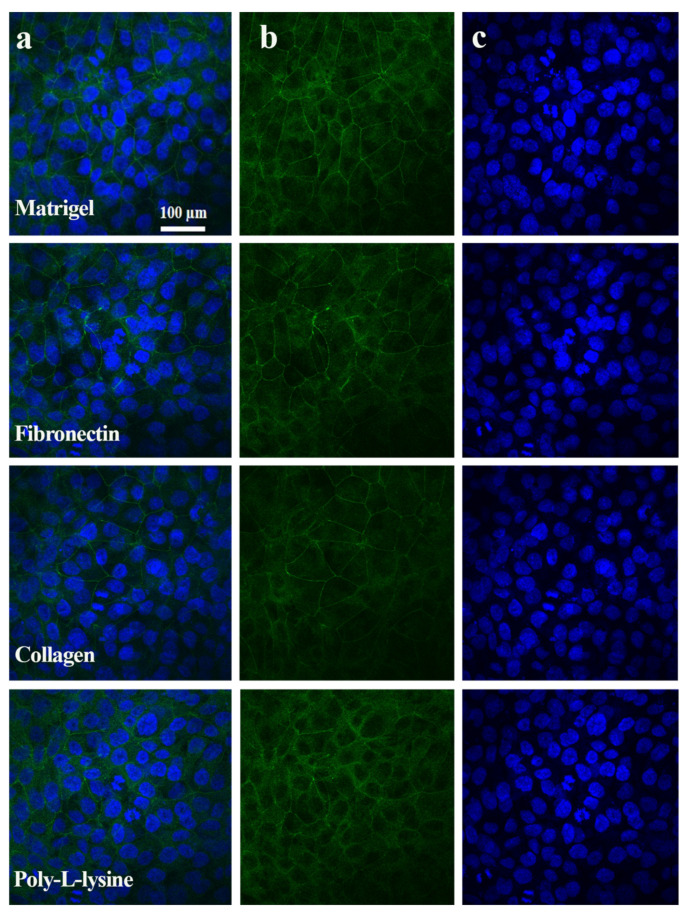
ZO-1 expression analysis in different ECM substrata. (**a**)Merge results of Zo-1 protein and nucleus staining image for Matrigel, fibronectin, collagen, and poly-L-lysine. The images were obtained after 6 days of liver microphysiological environmental culture. (**b**) The green color indicates ZO-1 expression in different ECM coated glass chip results (**c**) Blue color indicates the nuclei of cells. Scale bar: 100 μm.

**Figure 6:**
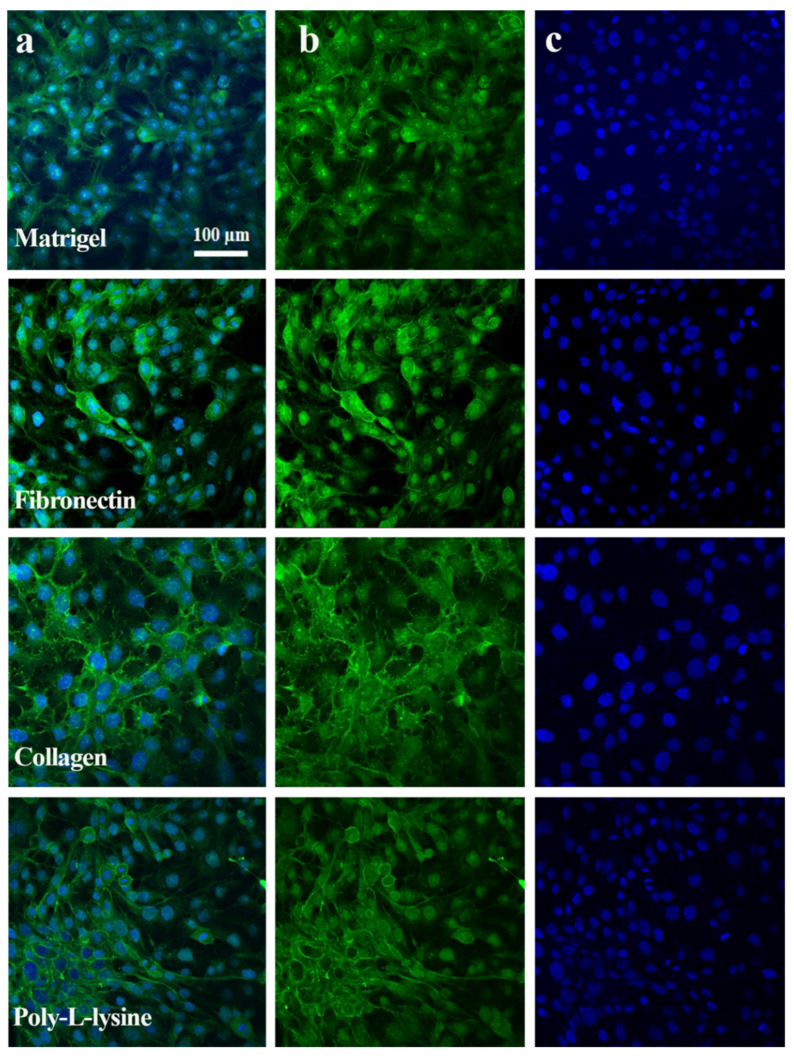
Expression of E-cadherin protein immunostaining in HepG2 cell line after 6 days of experiments with a microfluidic culture. (**a**) Merged results of tight junction protein expression, E-cadherin (green), and DAPI (blue) for nucleus staining with Matrigel, Fibronectin, Collagen, and Poly-L-Lysine based surface modified glass chip. (**b**) Singular expression of E-cadherin protein shown in green color in different ECM types above mentioned (**c**) Blue color indicates nuclei staining with DAPI. Scale bar: 100 μm.

TJPs maintain equilibrium between the intracellular and extracellular microenvironment by linking cells to other cells or attachment surfaces. Hepatic TJP expression changes drastically in response to drug exposure, cytokines, and inflammation [30]. Cellular barrier integrity is one of the most desired features of an MPS [31]. Previous MPS studies did not focus on TJP expression with respect to ECM types. The influence of different ECMs on ZO-1 and E-cadherin expression was examined through immunostaining, as shown in Figure 5 and Figure 6. The liver MPS was set up for 6 days, and the formation of the monolayers was observed. LabVIEW-based software was developed to analyze the immunofluorescence images based on the green light intensity, as shown in Figure S3 with an overview of the image processing and a detailed view is shown in Figures S4–S7.

**Figure 7:**
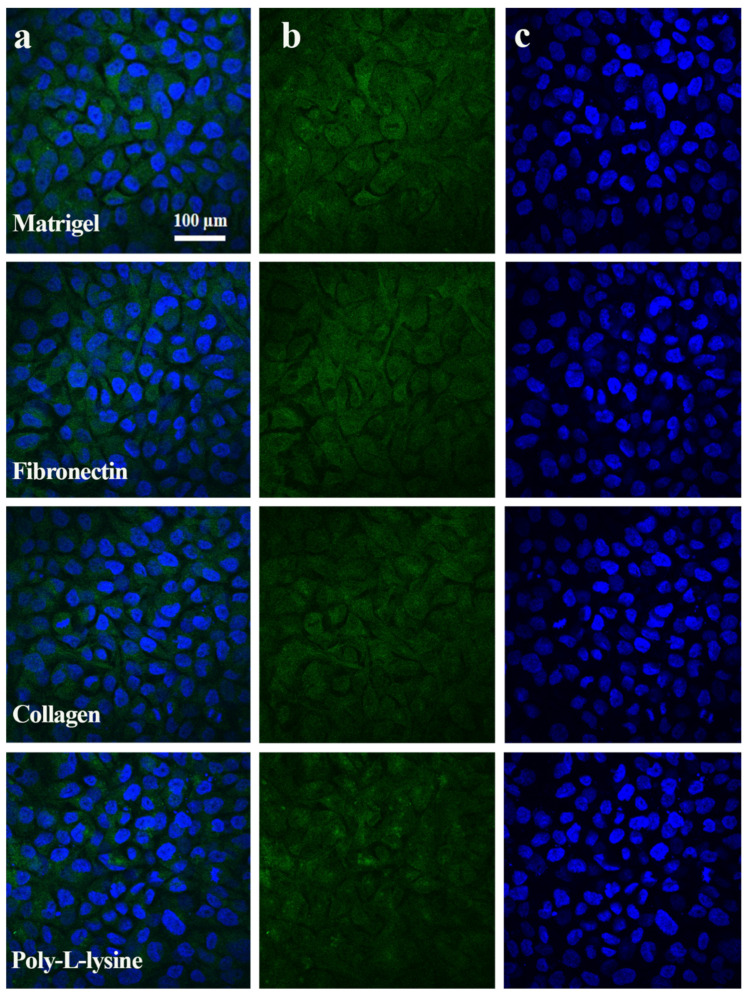
Albumin immunostaining images obtained after 6 days of hepatocytes culture performed using Matrigel, fibronectin, collagen, and poly-L-lysine, substrata respectively. (**a**) Combined figure of albumin and nuclei staining of hepatocyte cell culture with different ECM types. (**b**) Green color indicates albumin expression in hepatocyte in different ECM type coated glass chips. (**c**) DAPI was used for staining the nuclei represented in blue. Scale bar: 100 μm.

**Figure 8:**
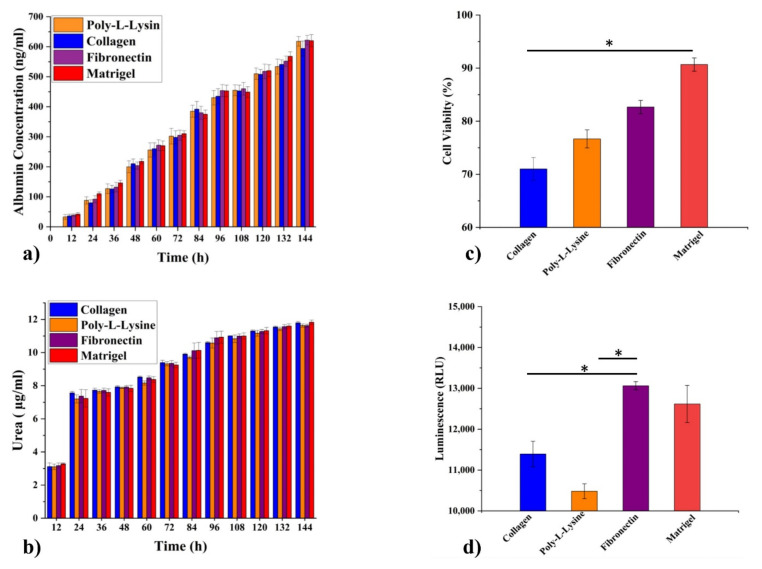
Molecular biomarker measurement and cell viability (live/dead assay) results for different ECMs in microphysiological system. (**a**) Albumin concentration under poly-L-lysine, collagen, fibronectin, and Matrigel. (**b**) Urea measurement in the HepG2 cell line cultured with poly-L-lysine, collagen, fibronectin, and Matrigel. (**c**) Live/dead assay (cell viability) measurement of HepG2 cell line cultured on different ECM-coated glass surfaces was performed after finishing the experiment and viability was calculated using ImageJ. (**d**) CYP3A4 activity assay of HepG2 cell line grown under dynamic culture conditions including different ECM types. Data are shown as mean ± SEM. * *p* ≤ 0.05.

The fluorescence of tight junction proteins, albumin, and live/dead assay immunostaining was analyzed with green, red, and blue colors. The green color showed the positive expression of TJPs and albumin (Figure 5, Figure 6 and Figure 7) and cell viability staining (Figure 3). In Figure 8c, the cell viability of the Matrigel is higher as compared to collagen, however, fibronectin cell viability did not show a significant difference to Matrigel. The obtained numeric values were mapped in 2D ranging from 0 to 255 for green color. Consequently, the green color magnitude determined the expression level of the respective biomarkers. Matrigel and fibronectin showed a well-defined monolayer formation, whereas the collagen- and poly L-lysine-based liver MPS showed areas with washed or detached cells. Likewise, a significant difference was observed in terms of TJP expression with different ECMs. The liver MPS based on Matrigel, and fibronectin showed better TJP expression than that based on collagen and poly-L-lysine. Hence, it has been proven that the extracellular matrix composition directly influences TJP formation, expression, and overall tissue barrier integrity [32].

### 3.6. Functional Biomarker Estimation

The functional biomarkers for hepatocytes mainly include albumin, urea, and CYP450 for their immediate upregulation or downregulation [33]. The expression profile of functional biomarkers was evaluated to assess the impact of different ECMs on biomarker secretion. Cell culture medium samples were collected after every 12 h throughout the experiment to quantify albumin and urea. A steady increase in albumin and urea release was observed, indicating normal physiological conditions, as shown in Figure 8a,b. There was no significant difference in albumin synthesis in the liver MPS based on different ECM types. It can be inferred that ECM composition does not directly affect the albumin and urea release of hepatocytes in a dynamic cell culture environment. It has been found that intracellular albumin has superior diagnostic value, and its concentration may differ for extracellular albumin [34]. To determine the impact of ECM composition on intracellular albumin expression, hepatocytes were stained for intracellular localization of albumin via immunofluorescence staining. A significant difference was recorded among the liver MPSs based on different ECMs. Matrigel-based liver MPS hepatocytes showed the highest cytosolic albumin localization compared to other ECM-based liver MPSs. However, the release of albumin and urea showed a consistent increase with time. Additionally, a CYP3A4 chemiluminescence assay was performed at the termination of the experiment. CYP3A4 is one of the subenzymes of the cytochrome P450 enzyme of hepatocytes and plays a crucial role in drug metabolism [35]. The results indicated an insignificant difference in the concentration of CYP3A4 from different liver MPSs (Figure 8d). Fibronectin showed the highest luminescence compared to poly-L-lysine, which showed the lowest CYP3A4 activity. In Figure 8c, Matrigel shows significant viability as compared to other ECM candidates while, with respect to CYP450 metabolic activity, fibronectin exhibited a significant activity in comparison with poly-L-lysine and collagen.

Hepatocytes are the major building blocks, making up to approximately 80% of the liver, and are critical for necessary metabolic and secretory functions in response to drug treatment and present great potential for drug development pipelines [36,37]. Moreover, the liver inflammation contributed by different liver cell types proceeds to multiple disease etiologies, eventually causing hepatocellular carcinoma [38,39,40]. The current study focuses on HepG2 cell line-based analysis of hepatocytes’ capacity for tissue formation. Although the limitations of the study include the absence of other cell types from liver lineages, i.e., stellate cells, liver sinusoidal endothelial cells, and Kupffer cells, and RNA-based expression analysis of ECM precursors, i.e., α-SMA, fibronectin, and collagen, the study presents substantial insight into the role of singular ECM components in comparison with a commercial mixture (Matrigel^TM^). The morphological and metabolic indications suggest that fibronectin presents nearly similar tissue formation potential to Matrigel.

It can be inferred that the influence of ECMs on molecular biomarkers for cellular growth was unreliable owing to their relevance to tissue formation. Image thresholding analysis using Fiji^TM^ and an image processing tool by LabVIEW have the potential to improve the overall assessment of tissue formation. Albumin ELISA and a urea assay showed no significant difference among the diversity of ECM concentrations but a slight variation in the result. The albumin staining image processed by LabVIEW tool also showed comparable results to that of conventional ELISA experiments. However, the MPS overall uses less media, and image processing tools can be used for biomarker analysis with better predictability of the results. Furthermore, LabVIEW utilized TJP expression and live/dead assay evaluations using image processing and provided tangible evidence of tissue confluency for better emulation of human physiology (Figure S8).

## 4. Conclusions

Natural and semi-synthetic biocompatible materials function as anchoring substrates for in vitro cell culture in OoC platforms. ECM is a crucial element for tissue development and cellular attachment via TJPs. In addition, image analysis tools present an opportunity to process and classify the data to better understand tissue development and predict the fate of MPSs. Based on implementation of a polynomial regression model in the cell attachment, image thresholding data in cell differentiation, and intensity-dependent image analysis results, we can enrich the prediction of biomarker secretion data of tissue microenvironments. Upon analyzing TJP expression profiles, we found that Matrigel and fibronectin are the most relevant for liver MPSs due to their support of better tissue growth and adherence. Collagen and poly-L-lysine can also be used, but they provide a less suitable physiological microenvironment than Matrigel and fibronectin. Moreover, fibronectin supports physiologically relevant metabolism and morphology of hepatocytes and, simultaneously, it presents a cost-effective solution as an alternative to Matrigel. Different ECM components cause considerable differences in cell adhesion, biomarker production, growth rate, morphology, and TJP expression. The choice of the most relevant ECM enhances the differentiation capacity of cells to retain their phenotype in an MPS. Additionally, this could result in better output from cell-based biological assays and permit improved translation from in vivo to in vitro models for disease and drug analysis.

## Figures and Tables

**Table 1:** Different ECM concentration and percentage of area of attachment results from image processing.

Matrigel		Fibronectin		Collagen		Poly-L-Lysine	
Applied Concentration	% Area of Cell Attachment	Applied Concentration	% Area of Cell Attachment	Applied Concentration	% Area of Cell Attachment	Applied Concentration	% Area of Cell Attachment
100 µg/mL	80.371	10 µg/mL	73.468	100 µg/mL	43.268	2 µg/mL	63.818
125 µg/mL	80.649	13 µg/mL	78.364	125 µg/mL	45.523	3 µg/mL	65.485
150 µg/mL	81.917	15 µg/mL	84.995	150 µg/mL	47.887	5 µg/mL	70.124
175 µg/mL	88.793	20 µg/mL	84.998	175 µg/mL	50.123	6 µg/mL	70.32
200 µg/mL	91.539	25µg/mL	85.523	200 µg/mL	58.867	7 µg/mL	70.522
R^2^ = 0.9477, RMSE = 1.3260		R^2^ = 0.9168, RMSE = 1.7098		R^2^ = 0.9670, RMSE = 1.2399		R^2^ = 0.9794, RMSE = 0.5192	

**Table 2:** Different ECM concentration confirmation data of cell attachment. Prediction of cell attachment percentage of area and experimental area of cell attachment data before starting a dynamic culture condition and prediction error.

Material	Coefficient	Applied Concentration	Prediction of Area of Cell Attachment (%)	Actual Area of Cell Attachment (%)	Prediction Error (%)
Matrigel	p1 = 0.001205 p2 = −0.2396 p3 = 91.97	120 µg/mL	80.57	79.253	1.662
Fibronectin	p1 = −0.1045 p2 = 4.426 p3 = 39.75	11 µg/mL	75.7915	78.283	3.183
Collagen	p1 = 0.001469 p2 = −0.2974 p3 = 58.86	130 µg/mL	45.0241	46.123	2.383
Poly-L-Lysine	p1 = −0.31 p2 = 4.217 p3 = 56.28	2.5 µg/mL	64.885	68.283	4.976

## Data Availability

The data presented in this study are available on request from the corresponding author.

## Supplementary Materials

The following are available online at https://www.mdpi.com/article/10.3390/polym13173016/s1, Figure S1. Preparation of the Seeding kit for ECM coating, cell seeding and staining, Figure S2. Image analysis results obtained using Fiji 2020 software, Figure S3. Graphical user interface of the LABVIEW based image processing tool overview, Figure S4. ZO-1 staining for tight junction proteins expression analysis by image processing, Figure S5. Albumin staining-based image analysis for Matrigel, fibronectin, collagen, and poly-l-lysine in LabVIEW, Figure S6. E-Cadherin staining for tight junction proteins expression analysis by image processing, Figure S7. Cell viability (live/dead assay) analysis in the Matrigel, fibronectin, collagen and poly-l-lysine analysis used by LabVIEW software, Figure S8. (A) Fluorescently stained images were analyzed using LabVIEW software, Figure S9. TEER graphs for hepatocyte dynamic microenvironment culture results with Matrigel, Fibronectin, Collagen and Poly-L-Lysine, Figure S10. Polynomial Regression Coefficient Results.

## References

[1] Jaberi A., Esfahani A.M., Aghabaglou F., Park J.S., Ndao S., Tamayol A., Yang R. (2020). Microfluidic Systems with Embedded Cell Culture Chambers for High-Throughput Biological Assays. ACS Appl. Bio Mater..

[2] Gauvin R., Chen Y.-C., Lee J.W., Soman P., Zorlutuna P., Nichol J.W., Bae H., Chen S., Khademhosseini A. (2012). Microfabrication of complex porous tissue engineering scaffolds using 3D projection stereolithography. Biomaterials.

[3] Kim B.S., Das S., Jang J., Cho D.-W. (2020). Decellularized Extracellular Matrix-based Bioinks for Engineering Tissue- and Organ-specific Microenvironments. Chem. Rev..

[4] Hansen N., Genovese F., Leeming D., Karsdal M. (2015). The importance of extracellular matrix for cell function and in vivo likeness. Exp. Mol. Pathol..

[5] Fitzpatrick L.E., McDevitt T.C. (2015). Cell-derived matrices for tissue engineering and regenerative medicine applications. Biomater. Sci..

[6] Mastrangeli M., Millet S., Mummery C., Loskill P., Braeken D., Eberle W., Cipriano M., Fernandez L., Graef M., Gidrol X. (2019). Building blocks for a European Organ-on-Chip roadmap. ALTEX-Altern. Anim. Exp..

[7] Reyes D.R., van Heeren H., Guha S., Herbertson L., Tzannis A.P., Ducrée J., Bissig H., Becker H. (2020). Accelerating innovation and commercialization through standardization of microfluidic-based medical devices. Lab A Chip.

[8] Salih A.R.C., Farooqi H.M.U., Kim Y.S., Lee S.H., Choi K.H. (2020). Impact of serum concentration in cell culture media on tight junction proteins within a multiorgan microphysiological system. Microelectron. Eng..

[9] Kang T., Park C., Meghani N., Tran T.T., Tran P.H., Lee B.-J. (2020). Shear Stress-Dependent Targeting Efficiency Using Self-Assembled Gelatin–Oleic Nanoparticles in a Biomimetic Microfluidic System. Pharmaceutics.

[10] Meghani N., Kim K.H., Kim S.H., Lee S.H., Choi K.H. (2020). Evaluation and live monitoring of pH-responsive HSA-ZnO nanoparticles using a lung-on-a-chip model. Arch. Pharmacal. Res..

[11] Meghani N.M., Amin H., Park C., Cui J.-H., Cao Q.-R., Choi K.H., Lee B.-J. (2020). Combinatory interpretation of protein corona and shear stress for active cancer targeting of bioorthogonally clickable gelatin-oleic nanoparticles. Mater. Sci. Eng. C.

[12] Sart S., Yan Y., Li Y., Lochner E., Zeng C., Ma T. (2016). Crosslinking of extracellular matrix scaffolds derived from pluripotent stem cell aggregates modulates neural differentiation. Acta Biomater..

[13] Vasudevan S., Kajtez J., Bunea A.-I., Gonzalez-Ramos A., Ramos-Moreno T., Heiskanen A., Kokaia M., Larsen N.B., Martínez-Serrano A., Keller S.S. (2019). Leaky Optoelectrical Fiber for Optogenetic Stimulation and Electrochemical Detection of Dopamine Exocytosis from Human Dopaminergic Neurons. Adv. Sci..

[14] Rosso F., Giordano A., Barbarisi M., Barbarisi A. (2004). From cell–ECM interactions to tissue engineering. J. Cell. Physiol..

[15] Ali M., Kim Y.S., Khalid M.A.U., Soomro A.M., Lee J.-W., Lim J.-H., Choi K.H., Ho L.S. (2020). On-chip real-time detection and quantification of reactive oxygen species in MCF-7 cells through an in-house built fluorescence microscope. Microelectron. Eng..

[16] Soomro A.M., Jabbar F., Ali M., Lee J.-W., Mun S.W., Choi K.H. (2019). All-range flexible and biocompatible humidity sensor based on poly lactic glycolic acid (PLGA) and its application in human breathing for wearable health monitoring. J. Mater. Sci. Mater. Electron..

[17] Soomro A.M., Khalid M.A.U., Shah I., Kim S.w., Kim Y.S., Choi K.H. (2020). Highly stable soft strain sensor based on Gly-KCl filled sinusoidal fluidic channel for wearable and water-proof robotic applications. Smart Mater. Struct..

[18] Soomro A.M., Memon F.H., Lee J.-W., Ahmed F., Kim K.H., Kim Y.S., Choi K.H. (2021). Fully 3D printed Multi-Material Soft Bio-Inspired Frog for Underwater Synchronous Swimming. Int. J. Mech. Sci..

[19] Kausar F., Farooqi M.-A., Farooqi H.-M.-U., Salih A.-R.-C., Khalil A.-A.-K., Kang C.-w., Mahmoud M.H., Batiha G.-E.-S., Choi K.-h., Mumtaz A.-S. (2021). Phytochemical Investigation, Antimicrobial, Antioxidant and Anticancer Activities of Acer cappadocicum Gled. Life.

[20] Lee H., Chae S., Kim J.-Y., Han W., Kim J., Choi Y., Cho D.-W. (2019). Cell-printed 3D liver-on-a-chip possessing a liver microenvironment and biliary system. Biofabrication.

[21] Ehrlich A., Duche D., Ouedraogo G., Nahmias Y. (2019). Challenges and opportunities in the design of liver-on-chip microdevices. Annu. Rev. Biomed. Eng..

[22] Farooqi H.M.U., Khalid M.A.U., Kim K.H., Lee S.R., Choi K.H. (2020). Real-time physiological sensor-based liver-on-chip device for monitoring drug toxicity. J. Micromech. Microeng..

[23] Lee K., Murugesan M., Lee S.-M., Kang B.-S. (2017). A comparative study on Arrhenius-type constitutive models with regression methods. Trans. Mater. Process..

[24] Murugesan M., Kang B.-S., Lee K. (2015). Multi-objective design optimization of composite stiffened panel using response surface methodology. Compos. Res..

[25] Asif A., Kim K.H., Jabbar F., Kim S., Choi K.H. (2020). Real-time sensors for live monitoring of disease and drug analysis in microfluidic model of proximal tubule. Microfluid. Nanofluidics.

[26] Khalid M.A.U., Kim Y.S., Ali M., Lee B.G., Cho Y.-J., Choi K.H. (2020). A lung cancer-on-chip platform with integrated biosensors for physiological monitoring and toxicity assessment. Biochem. Eng. J..

[27] Urbanczyk M., Layland S.L., Schenke-Layland K. (2020). The role of extracellular matrix in biomechanics and its impact on bioengineering of cells and 3D tissues. Matrix Biol..

[28] Henry O.Y.F., Villenave R., Cronce M.J., Leineweber W.D., Benz M.A., Ingber D.E. (2017). Organs-on-chips with integrated electrodes for trans-epithelial electrical resistance (TEER) measurements of human epithelial barrier function. Lab A Chip.

[29] Farooqi H.M.U., Kang B., Khalid M.A.U., Salih A.R.C., Hyun K., Park S.H., Huh D., Choi K.H. (2021). Real-time monitoring of liver fibrosis through embedded sensors in a microphysiological system. Nano Converg..

[30] Han X., Fink M.P., Uchiyama T., Yang R., Delude R.L. (2004). Increased iNOS activity is essential for hepatic epithelial tight junction dysfunction in endotoxemic mice. Am. J. Physiol. Gastrointest. Liver Physiol..

[31] Tang H., Abouleila Y., Si L., Ortega-Prieto A.M., Mummery C.L., Ingber D.E., Mashaghi A. (2020). Human organs-on-chips for virology. Trends Microbiol..

[32] Gissen P., Arias I.M. (2015). Structural and functional hepatocyte polarity and liver disease. J. Hepatol..

[33] Asif A., Park S.H., Soomro A.M., Khalid M.A.U., Salih A.R.C., Kang B., Ahmed F., Kim K.H., Choi K.H. (2021). Microphysiological system with continuous analysis of albumin for hepatotoxicity modeling and drug screening. J. Ind. Eng. Chem..

[34] Seidkhani-Nahal A., Allameh A., Soleimani M. (2019). Antioxidant and reactive oxygen species scavenging properties of cellular albumin in HepG2 cells is mediated by the glutathione redox system. Biotechnol. Appl. Biochem..

[35] Lu X., Li Y., Thunders M., Cavanagh J., Matthew C., Wang X., Zhou X., Qiu J. (2017). Differential protein expression and localization of CYP450 enzymes in three species of earthworm; is this a reflection of environmental adaptation?. Chemosphere.

[36] Klover P.J., Mooney R.A. (2004). Hepatocytes: Critical for glucose homeostasis. Int. J. Biochem. Cell Biol..

[37] Gomez-Lechon M., Donato M., Castell J., Jover R. (2003). Human hepatocytes as a tool for studying toxicity and drug metabolism. Curr. Drug Metab..

[38] Asif A., Khalid M., Manzoor S., Ahmad H., Rehman A.U. (2019). Role of purinergic receptors in hepatobiliary carcinoma in Pakistani population: An approach towards proinflammatory role of P2X4 and P2X7 receptors. Purinergic Signal..

[39] Ponziani F.R., Bhoori S., Castelli C., Putignani L., Rivoltini L., Del Chierico F., Sanguinetti M., Morelli D., Sterbini F.P., Petito V. (2019). Hepatocellular carcinoma is associated with gut microbiota profile and inflammation in nonalcoholic fatty liver disease. Hepatology.

[40] Imai Y., Yoshida O., Watanabe T., Yukimoto A., Koizumi Y., Ikeda Y., Tokumoto Y., Hirooka M., Abe M., Hiasa Y. (2019). Stimulated hepatic stellate cell promotes progression of hepatocellular carcinoma due to protein kinase R activation. PLoS ONE.

