# Supplementary material for: Extracellular Matrix Optimization for Enhanced Physiological Relevance in Hepatic Tissue-Chips
Source: Polymers (Basel). 2021 Sep 6;13(17):3016. doi: 10.3390/polym13173016 (PMC8434375; doi:10.3390/polym13173016)
Supplement: Supplementary file 1 [file polymers-13-03016-s001.zip › polymers-1365181-supplementary.pdf]

Supplementary Information

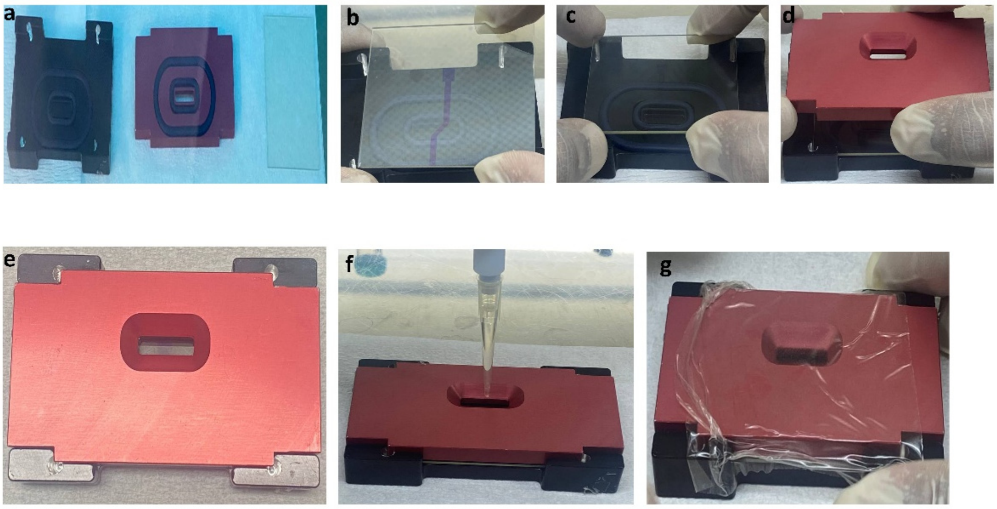

Figure S1. Preparation of the Seeding kit for ECM coating, cell seeding and staining. a. UV sterilization of the sending kit, b-d. chip placement and assembly. f. ECM coating/seeding of the cells, g. membrane cover.

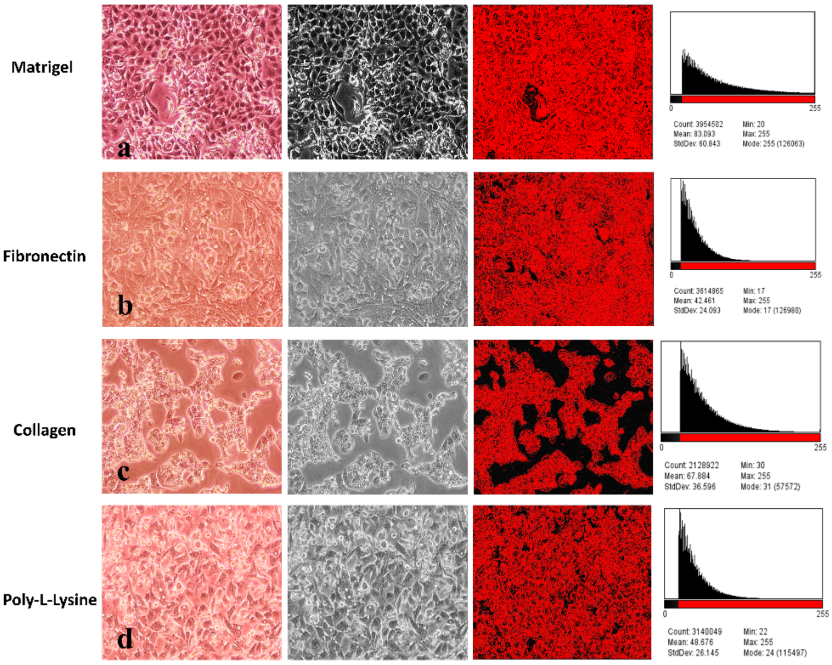

Figure S2. Image analysis results obtained using Fiji 2020 software. (a) Cell image with Matrigel, images from left to right include; real light microscopic image, black &white image, image thresholding result and histogram picture (b) Cell image with fibronectin, images from left to right include; real light microscopic image, black &white image, image thresholding result and histogram picture (c) Cell image with collagen-I, images from left to right include; real light microscopic image, black &white image, image thresholding result and histogram picture (d) Cell image with poly-L-lysine, images from left to right include; real light microscopic image, black &white image, image thresholding result and histogram picture

Figure S3. Graphical user interface of the LABVIEW based image processing tool overview.

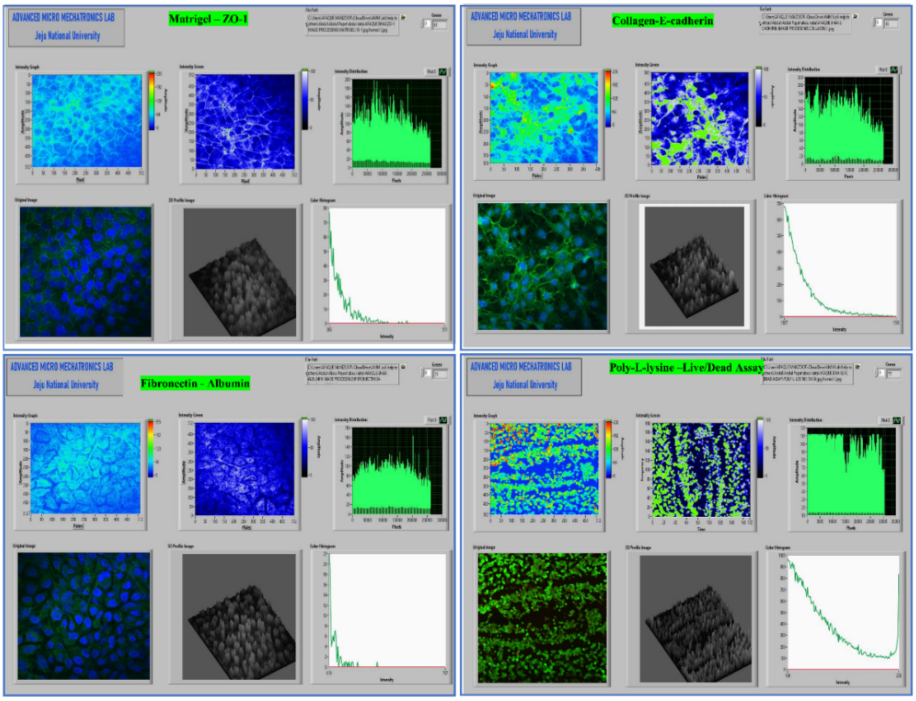

Figure S4. ZO-1 staining for tight junction proteins expression analysis by image processing.

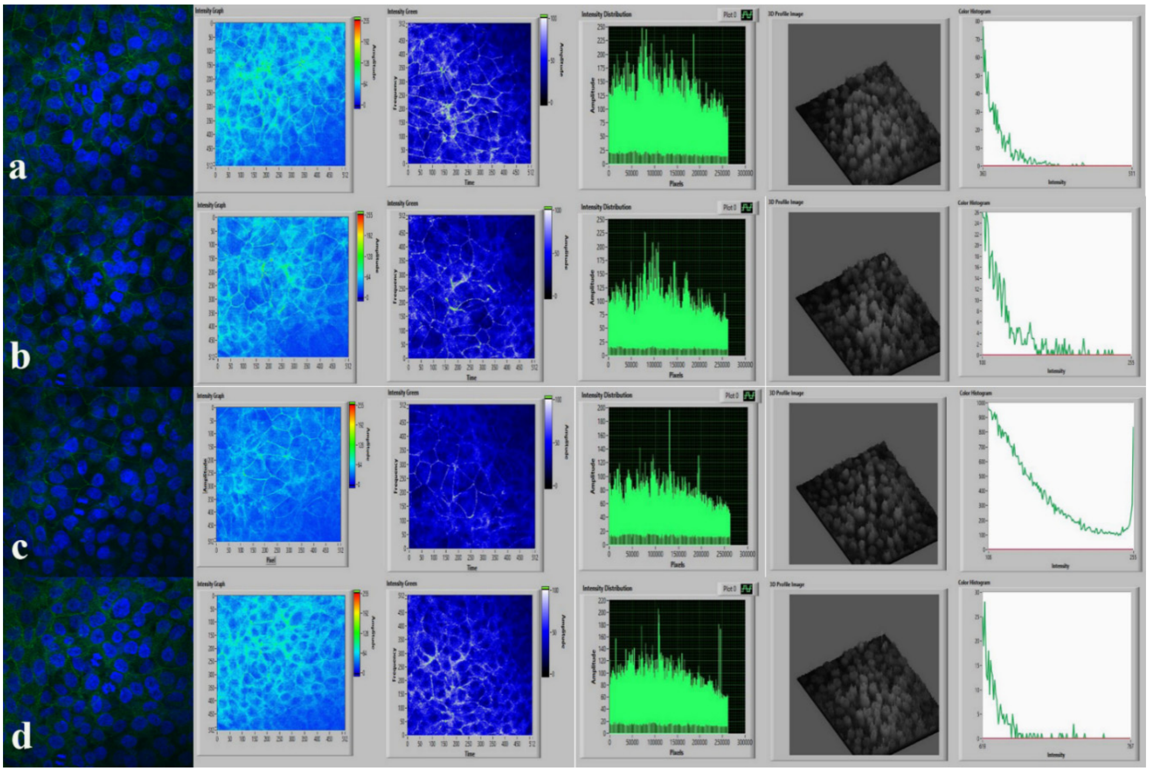

LabVIEW based graphical user interface was implemented to analyze Matrigel, fibronectin, collagen, and poly-l-lysine impact on tissue formation. (a) Matrigel confocal image, ZO-1 total intensity graph, green intensity of ZO-1, green intensity distribution, 3d profile image generation image and color histogram intensity graph (from left to right). (b) Fibronectin confocal image. ZO-1 total intensity graph, green intensity of ZO-1, green intensity distribution, 3d profile image generation image and color histogram intensity graph (from left to right). (c) Collagen confocal image, ZO-1 total intensity graph, green intensity of ZO-1, green intensity distribution, 3d profile image generation image and color histogram intensity graph (from left to right). (d) Poly-l-lysine confocal image, ZO-1 total intensity graph, green intensity of ZO-1, green intensity distribution, 3d profile image generation image and color histogram intensity graph (from left to right).

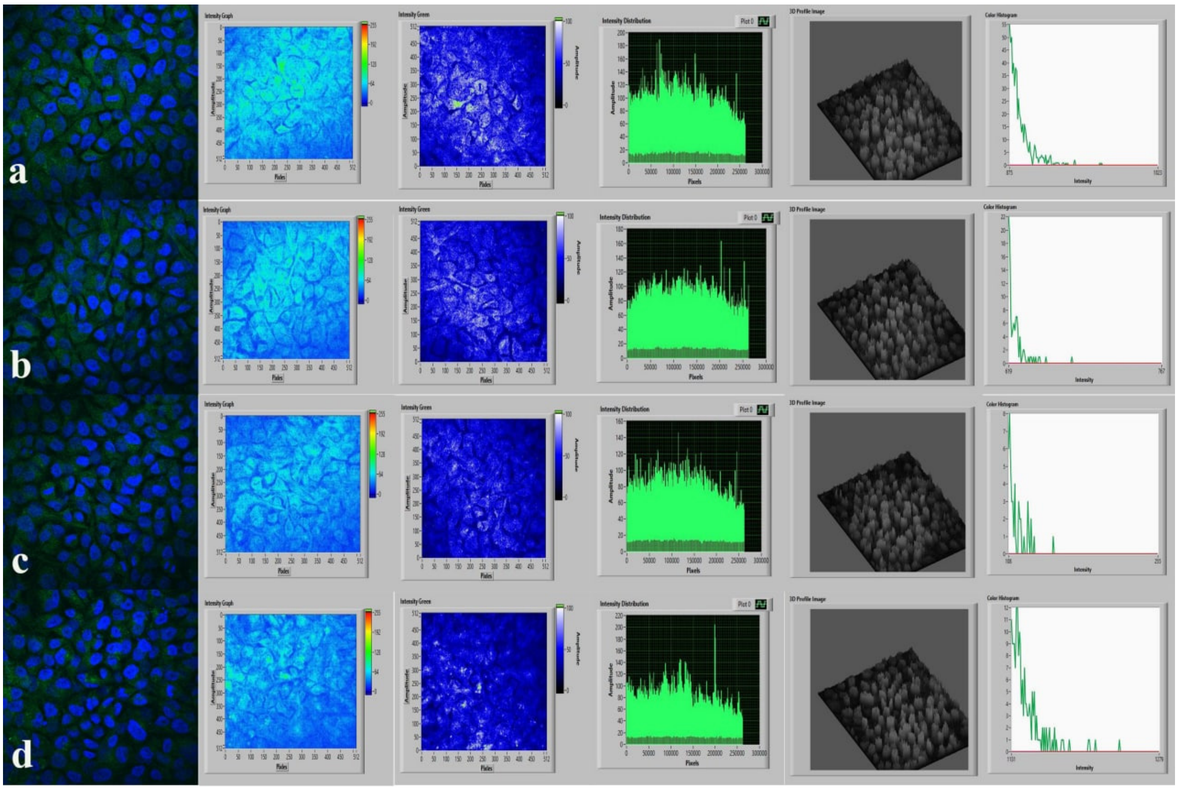

Figure S5. Albumin staining-based image analysis for Matrigel, fibronectin, collagen, and poly-l-lysine in LabVIEW (a) Matrigel confocal image, albumin total intensity graph, green intensity of albumin, green intensity distribution, 3d profile image generation image and color histogram intensity graph (from left to right). (b) Fibronectin confocal image, albumin total intensity graph, green intensity of albumin, green intensity distribution, 3d profile image generation image and color histogram intensity graph (from left to right). (c) Collagen confocal image, albumin total intensity graph, green intensity of albumin, green intensity distribution, 3d profile image generation image and color histogram intensity graph (from left to right). (d) Poly-l-lysine confocal image, albumin total intensity graph, green intensity of albumin, green intensity distribution, 3d profile image generation image and color histogram intensity graph (from left to right).

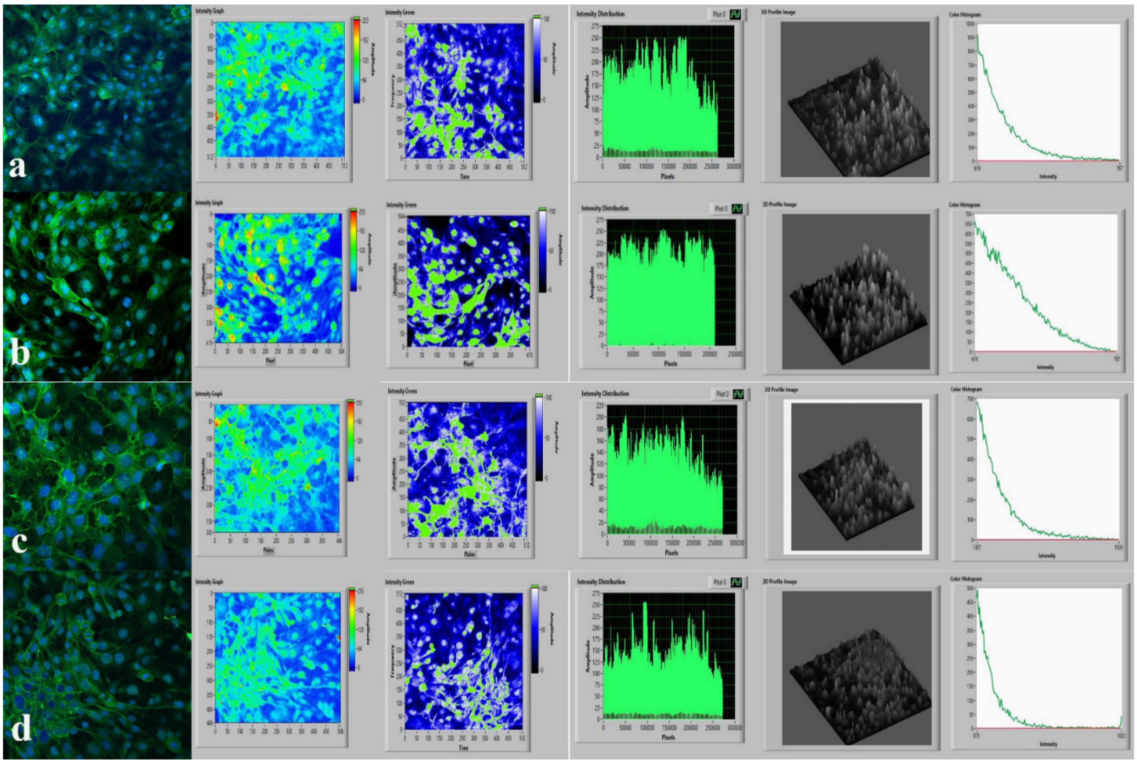

Figure S6. E-Cadherin staining for tight junction proteins expression analysis by image processing. LabVIEW based graphical user interface was implemented to analyze Matrigel, fibronectin, collagen, and poly-l-lysine impact on tissue formation. (a) Matrigel confocal image. E-cadherin total intensity graph, Green intensity of E-cadherin, green intensity distribution, 3D profile image generation image and color histogram Intensity graph (from left to right) (b) Fibronectin confocal image. E-cadherin total intensity graph, green intensity of e-cadherin, green intensity distribution, 3d profile image generation image and color histogram intensity graph (from left to

right) (c) Collagen confocal image. E-cadherin total intensity graph, green intensity of e-cadherin, green intensity distribution, 3d profile image generation image and color histogram intensity graph (from left to right) (d) Poly-l-lysine confocal image. E-cadherin total intensity graph, green intensity of e-cadherin, green intensity distribution, 3d profile image generation image and color histogram intensity graph (from left to right)

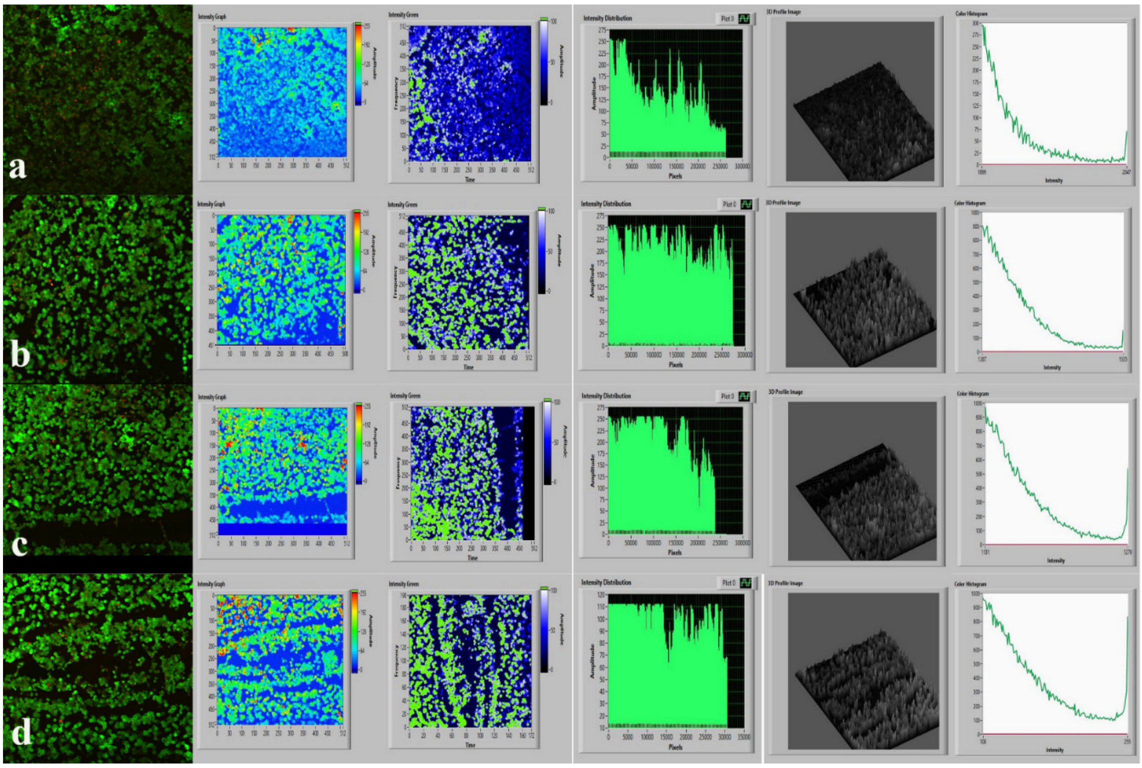

Figure S7. Cell viability (live/dead assay) analysis in the Matrigel, fibronectin, collagen and poly-l-lysine analysis used by LabVIEW software. (a) Confocal image of the cell viability in Matrigel. Total intensity graph, green intensity of calcein am, green intensity distribution, 3d profile image generation image and color histogram intensity graph (from left to right) (b) Confocal image of the cell viability in fibronectin. Total intensity graph, green intensity of calcein am, green intensity distribution, 3d profile image generation image and color histogram intensity graph (from left to right). (c) Confocal image of the cell viability in collagen. from left to right total intensity graph, green intensity of calcein am, green intensity distribution, 3d profile image generation image and color histogram intensity graph (from left to right) (d) Confocal image of the cell viability in poly-l-lysine. Total intensity graph, green intensity of calcein am, green intensity distribution, 3d profile image generation image and color histogram intensity graph (from left to right)

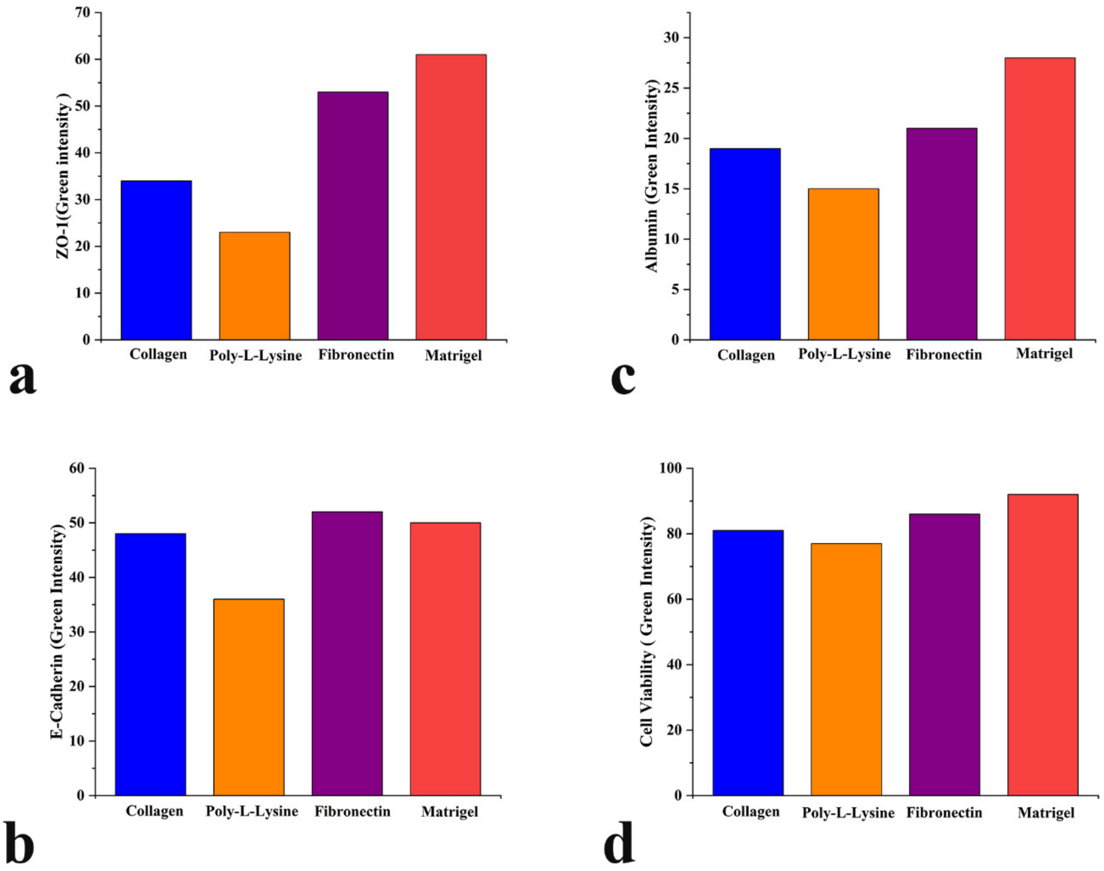

Figure S8. (A) Fluorescently stained images were analyzed using LabVIEW software. (a) ZO-1 tight junction protein green intensity measurement HepG2 cell line cultured using Poly-L-Lysine, Collagen, Fibronectin, and Matrigel. (b) E-Cadherin expression measurement from the HepG2 cell line cultured with Poly-L-Lysine, Collagen, Fibronectin, and Matrigel. (c) Albumin green intensity measurement of HepG2 cell line cultured on different ECM-coated glass surface (d) Cell viability (live/dead assay) of HepG-2 cell line cultured using different ECMs.

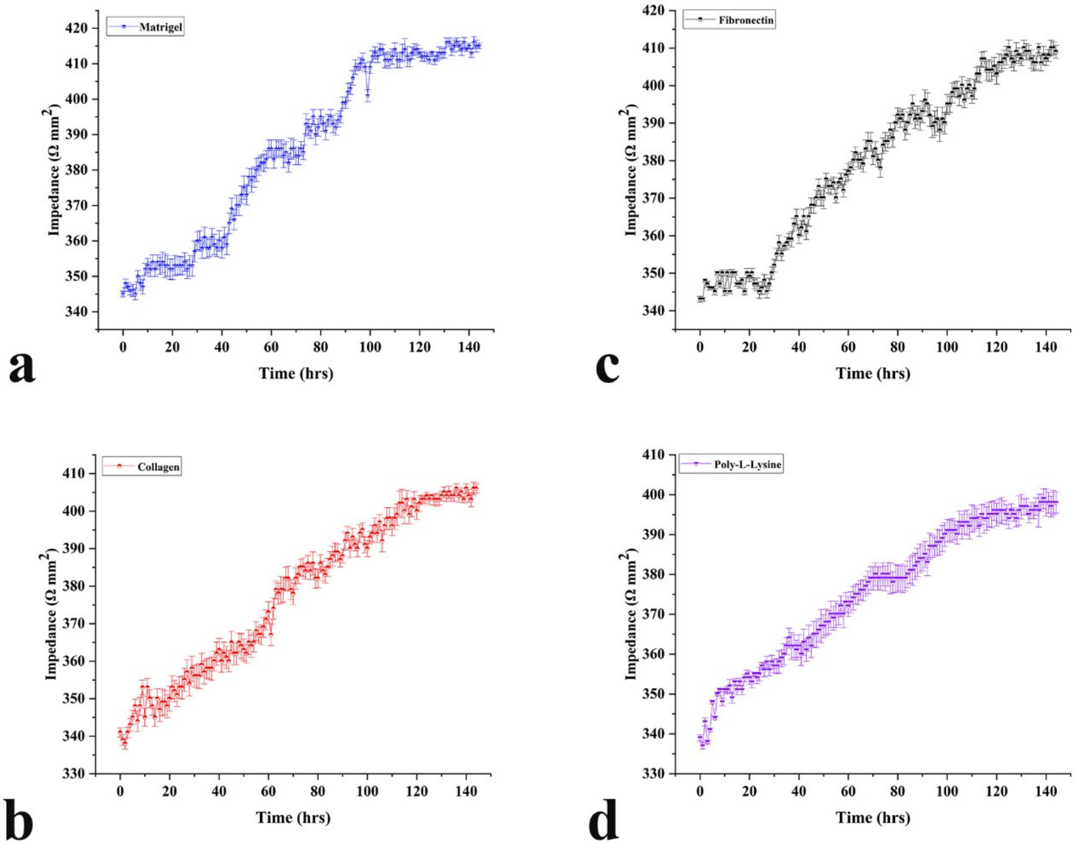

Figure S9. TEER graphs for hepatocyte dynamic microenvironment culture results with Matrigel, Fibronectin, Collagen and Poly-L-Lysine.

```
fitresult =

     Linear model Poly2:
     fitresult(x) = p1*x^2 + p2*x + p3
     Coefficients (with 95% confidence bounds):
       p1 =    0.001205  (-0.001864, 0.004274)
       p2 =     -0.2396  (-1.165, 0.6857)
       p3 =       91.97  (25.27, 158.7)
```

Matrigel

```
fitresult =

     Linear model Poly2:
     fitresult(x) = p1*x^2 + p2*x + p3
     Coefficients (with 95% confidence bounds):
       p1 =     -0.1045  (-0.2895, 0.08057)
       p2 =       4.426  (-2.151, 11)
       p3 =       39.75  (-14.21, 93.7)
```

Fibronectin

```
fitresult =

     Linear model Poly2:
     fitresult(x) = p1*x^2 + p2*x + p3
     Coefficients (with 95% confidence bounds):
       p1 =    0.001469  (-0.001373, 0.00431)
       p2 =     -0.2974  (-1.154, 0.5591)
       p3 =       58.86  (-2.889, 120.6)
```

Collagen

```
     Linear model Poly2:
     fitresult(x) = p1*x^2 + p2*x + p3
     Coefficients (with 95% confidence bounds):
       p1 =       -0.31  (-0.8265, 0.2065)
       p2 =       4.217  (-0.4313, 8.866)
       p3 =       56.28  (47.2, 65.36)
```

Poly-L-Lysine

Figure S10. Polynomial Regression Coefficient Results
